# Tobacco and Cancer

**Published:** 1886-06

**Authors:** 


### TOBACCO AND CANCER.

Dr. Crabtree of Boston, gives the following testimony on tobacco using:

"Is cigar smoking productive of cancer?"

The answer, by every physician of experience in mucus and blood diseases, must be in the affirmative. Among men tobacco using is almost the sole cause of cancer. I have had no little experience of this disease, and after nearly thirty years of careful observation I solemnly declare that I have never met with a case of cancer of the lip, tongue, throat or face that I did not trace to tobacco using—smoking mostly—as the cause. Cigar smoking was sometimes the only way these victims used tobacco. Catelain, the great Parisian caterer, died of smoker's cancer. Dr. Liza enumerates many cases in his experience where smoking tobacco produced cancer. But why go to France or England for evidence found at home? Where is our Senator Hill? or, worse, our General Grant. Why try to hide so notorious a fact that *cigar smoking killed our greatest general?* Dr. Douglas said "Smoking was the exciting cause of this cancer." I once had a case of a man with cancer of the lip just where he held his cigar. I induced him to leave off smoking while under treatment. After a few months a cure was seemingly effected, when he resumed the habit of smoking, followed by a return of the disease. Again he came to me, and we went over the same routine as before. After that he gave up the use of tobacco, and had no return of the disease. As so another case, a pipe smoker. The lip healed, and so remained till he returned to smoking, when the cancer returned. Not willing to give up his pipe, he went to a "cancer doctor," who applied a plaster, and "drew out the cancer," (Can you draw up a well?) Soon the cancer returned tenfold as malignant, drawn into the *wound*, of course, from all over the system, and the victim died in horrid disfigurement and agony."

"Certainly tobacco using in any form endangers the user to cancer. Snufftakers often have cancer of the nose and fauces. Dr. Barker (London) *British Medical Journal*, points to 75 cases of epithelioma, or what Buzenet calls "*plaques des fumeurs*," of whom 71 smoked, and in only one case of the 75 was there a hereditary predisposition to cancer. Yes, *cigar smoking is productive of cancer*."

---

**Young Doctor:** "They don't bleed people now-a-days as they did twenty years ago, do they, Professor?" Professor: "Not *with the lancet.*"

